# Health-related quality of life following ambulatory surgery procedures: assessment by RAND-36

**DOI:** 10.1186/1471-2253-12-30

**Published:** 2012-12-05

**Authors:** Kristiina Mattila, Merja Lahtela, Markku Hynynen

**Affiliations:** 1 Department of Anesthesiology and Intensive Care Medicine, Jorvi Hospital, Helsinki University Hospital, Espoo, Finland; 2 Department of Anesthesiology and Intensive Care Medicine, Lappland Central Hospital, Rovaniemi, Finland

**Keywords:** Quality of life, Outcome, Ambulatory surgery procedures, RAND-36

## Abstract

**Background:**

Increasing numbers of elective surgical procedures are performed as day-cases. The impact of ambulatory surgery on health-related quality of life in the recovery period has seldom been described.

**Methods:**

We assessed health-related quality of life in 143 adult outpatients scheduled for arthroscopic procedures of the knee and shoulder joints, laparoscopic cholecystectomy and inguinal hernia repair using the RAND 36-Item Health Survey preoperatively and one week after patients had returned to work or comparable normal daily routines.

**Results:**

Postoperatively all patient groups reported significant improvements in bodily pain and vitality. Physical functioning improved significantly in orthopedic and inguinal hernia patients. However, in the orthopedic groups, postoperative scores for physical health were still relatively lower compared to the general population reference values.

**Conclusions:**

Ambulatory surgery has a positive impact on health-related quality of life. Assessment of the recovery process is necessary for recognition of potential areas of improvement in care and postoperative rehabilitation.

## Background

Ambulatory surgery is considered the standard of elective operative care [1,2]. Cohort studies and clinical experience indicate that the practice is safe. Major morbidity is rare [3], discharge home is successful on the day of the operation [4,5], readmission to the hospital is seldom required, and overall patient satisfaction is high [6,7].

Improved quality of life is one of the main end-points following ambulatory surgery procedures. Patient-reported outcome measures comprise an essential part in the assessment of quality of surgical care and are recommended for benchmarking [8]. Quality of recovery in the early postoperative period has been assessed in several studies [9-12]. However, patient assessed recovery after the first postoperative week has been less frequently studied [13], and only few studies have evaluated health-related quality of life during the period, when patients are returning back to work and other daily routines [14-16].

Health-related quality of life describes the impact that health has on the individuals′ functional ability, physical, mental and social well-being [17]. The RAND 36-Item Health Survey and The Medical Outcomes Study Short Form-36 Health Survey are probably the most widely used, almost identical instruments for assessment of quality of life. They are generic profile measures, designed to be applicable to anyone, and to describe quality of life by creating a multidimensional profile of eight different health concepts [17].

The aim of this study was to evaluate the quality of postoperative recovery in ambulatory surgery patients undergoing four common outpatient procedures: inguinal hernia repair, laparoscopic cholecystectomy, and arthroscopic procedures of the knee or shoulder joints. Data was collected preoperatively and one week after the end of the sick leave, using the RAND-36 instrument. Postoperatively we expected to find an improvement in several dimensions of patient-reported quality of life. These findings are reported in the article.

## Methods

The study was an optional part of a prospective, cross-sectional, cohort study aimed to describe ambulatory surgery practice in Finland, carried out between February and April 2007 at 14 day surgery units [6]. Consecutive Finnish or Swedish speaking patients, aged 18 years or older scheduled for day-case inguinal hernia repair (Hernia group), laparoscopic cholecystectomy (LCC group), arthroscopic procedures of the knee (Knee group) or shoulder joints (Shoulder group) were asked to participate. The aim was to recruit 200 patients during the study period, 50 patients from each surgical group. Health-related quality of life was assessed pre- and postoperatively using the validated Finnish version of the RAND-36 questionnaire [18]. There were no changes in standard care during the study period. Ethical Committees of all participating hospitals accepted the approval given by the Ethical Committee of the Hospital District of Northern Savo.

Patients were recruited to the study at least one week before surgery by telephone or at a preoperative visit by anesthesiologists or nurses that were in charge of the study at different units. Patients gave their written consent after receiving oral and written information about the study protocol.

The preoperative questionnaire was returned on the day of the operation. The postoperative questionnaire was given to patients before discharge from the ambulatory surgery units with instructions to be filled-in and returned in a prepaid envelope one week after the end of the sick leave, or a comparable time. Only patients that returned both questionnaires were included in the analyses. Non-responding patients were not contacted.

Patient and procedure characteristics were documented on separate standardized sheets by nurses at the ambulatory surgery units. An extranet database was provided for the study by Intensium Ltd., a Finnish healthcare IT solution and service provider specialized in benchmarking.

The RAND-36 instrument is composed of 8 health dimensions including altogether 36 items. The 10-item dimension of *Physical Functioning* measures health-related limitations due to physical activities, the 4-item dimension of *Role Limitations Due to Physical Health Problems* measures the extent to which health interferes with working and other daily activities, the 2-item dimension of *Pain* measures pain frequency and its interference on health, the 5-item *General Health* dimension measures health in general, the 5-item dimension of *Emotional Well-being* measures general mood, depression, anxiety and well-being; the 3-item dimension of *Role Limitations Due to Emotional Problems* measures the extent to which emotional problems interfere with work and daily activities, the 2-item dimension of *Social Functioning* measures how health interferes with social activities, and the 4-item *Vitality* dimension measures how energetic or tired and worn out the patient feels. In addition one item inquires about the individuals` present health state compared to 12 months ago [17,18]. All items are scored from 0 to 100, with high scores indicating good quality of life and high level of functioning [18]. Of the 36 items, 20 are time-related. In the present study time-related items covered the preceding four weeks preoperatively and one week postoperatively.

The score for each health dimension is the mean value of the item scores included in it. According to general recommendations, mean dimension scores for each patient were calculated only when the minimum number of items required for each specific dimension were answered [18]. Results are expressed as means and standard deviations.

Intergroup comparisons between pre-and postoperative dimension scores were performed using the Wilcoxon test for two related samples. *P* values less than 0.05 were considered statistically significant. A change of at least six points in mean scale scores was considered clinically important. The estimate for clinically important difference was obtained from the literature [17-19]. According to expert opinion, a change of 6-10% on the breath of a relevant instrument scale, or a difference of 10 in median scores is considered clinically important.

## Results

**Figure 1:**
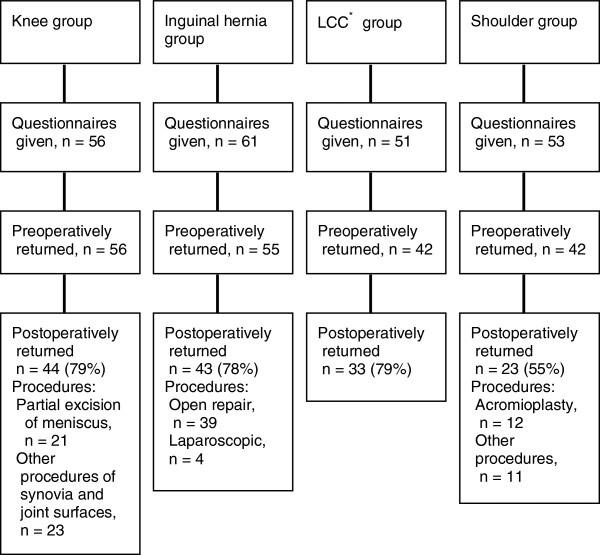
**Flow chart of patients recruited in the study.** The percentage of postoperatively returned questionnaires is given for the number of questionnaires that were returned preoperatively. *LCC = laparoscopic cholecystectomy.

Patients were recruited during a three month-period at 11 units. The study was introduced to 221 patients, of which 195 accepted to participate. Of these 143 (73%) returned the postoperative questionnaire and were included (Figure 1). Descriptive statistics of the patients and procedures are shown in Table 1. Median time for returning the postoperative questionnaires in the Knee, Hernia, Laparoscopic cholecystectomy and Shoulder groups were 31, 31, 23 and 66 days after surgery, respectively. In the preoperative questionnaires only 0.5%, and in the postoperative questionnaire 0.9% of items (n=5148) were unanswered.

**Table 1:** Demographic data and clinical information

	**Knee group**	**Hernia group**	LCC^* ^**group**	**Shoulder group**
	**(n = 44)**	**(n = 43)**	**(n = 33)**	**(n = 23)**
Age (yr), median (range)	54 (19 – 84)	54 (25 – 74)	51 (20 – 65)	49 (23 – 58)
Age group (yr)				
16 – 44	23	21	27	35
45 – 64	64	60	70	65
65 – 74	11	19	3	0
75 – 84	2	0	0	0
Sex (male/female)	45/55	91/9	24/76	70/30
ASA status^**^ (1/2/3)	50/50/0	42/49/9	61/36/3	61/35/4
Type of anesthesia				
General	12	28	100	61
Local		37		
Spinal	86	35		
Interscalne				39
Missing	2			
Operating surgeon				
Specialist/resident	68/32	72/28	85/15	91/9
Unplanned admission	2.3	9.3	18	17

Demographic data and clinical information of different study groups. Figures are shown as percentage (%), unless otherwise stated. ^*^LCC = laparoscopic cholecystectomy, ^**^ASA status = American Society of Anesthesiologists physical status classification system.

**Figure 2:**
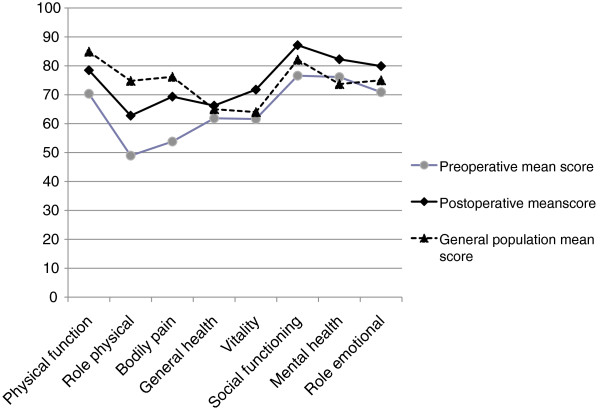
**RAND-36 dimension scores.** Scores for RAND-36 dimensions of health-related quality of life are shown for all study patients (n=143). General Finnish population scores are given for reference. Values are mean. Differences between preoperative and postoperative mean values are statistically significant for all dimensions: Role Emotional (*P < 0.05*), all others (*P <0.005*).

Figure 2 demonstrates the mean pre- and postoperative scores for the eight health dimensions in the combined study population. Overall, in the outpatient population the mean scores for physical functioning and bodily pain improved significantly but were postoperatively still slightly below the reference mean values of the general Finnish population (n=2060) [18]. Table 2 shows the mean pre-and postoperative dimension scores in each surgical group. Postoperatively the improvement in mean scores measuring bodily pain and vitality was clinically relevant and statistically significant in all patient groups. Physical functioning improved significantly in both orthopedic groups and in the Hernia group, while in the Laparoscopic cholecystectomy group physical activity remained at the same high level. Preoperatively the orthopedic patients experienced more limitations due to physical health problems than the other two patient groups. Orthopedic and laparoscopic cholecystectomy patients reported improved social functioning. The difference in pre –and postoperative scores for interference of emotional problems in daily activities was not statistically significant in any of the patient groups. None of the postoperative mean dimension scores decreased significantly compared to preoperative values.

**Table 2:** Preoperative and postoperative mean scores

		**Knee group**	**Hernia group**	**LCC**^**a **^**group**	**Shoulder group**
		**(n = 44)**	**(n = 43)**	**(n = 33)**	**(n = 23)**
Physical Function	Pre^**^	49.2 (29.5)	75.6 (20.0)	90.9 (13.1)	71.5 (17.5)
	Post^***^	64.4 (24.5)	83.6 (20.7)	89.7 (16.2)	79.7 (21.0)
	*P*	*0.001*	*0.01*	*NS*	*0.01*
Role Physical	Pre	31.3 (35.8)	55.2 (39.9)	74.0 (36.8)	36.2 (42.5)
	Post	56.8 (44.6)	64.5 (39.0)	68.9 (37.0)	62.0 (39.8)
	*P*	*0.000*	*NS*	*NS*	*0.01*
Bodily Pain	Pre	44.0 (25.6)	64.1 (24.8)	63.2 (24.5)	40.0 (25.5)
	Post	59.7 (24.2)	74.5 (23.6)	77.5 (21.6)	66.0 (28.0)
	*P*	*0.001*	*0.01*	*0.01*	*0.000*
General Health	Pre	58.8 (18.6)	63.7 (21.2)	64.7 (17.2)	60.2 (18.4)
	Post	60.4 (20.2)	67.1 (20.6)	72.9 (18.8)	66.3 (18.9)
	*P*	*NS*	*NS*	*0.004*	*0.02*
Vitality	Pre	56.0 (22.1)	68.7 (21.8)	65.9 (20.3)	52.8 (22.8)
	Post	64.7 (23.3)	77.7 (21.1)	77.0 (16.0)	66.4 (22.4)
	*P*	*0.02*	*0.002*	*0.000*	*0.01*
Social Functioning	Pre	67.6 (26.7)	84.9 (18.8)	80.5 (22.4)	72.7 (23.7)
	Post	82.8 (20.0)	88.4 (20.7)	91.3 (14.5)	87.0 (16.2)
	*P*	*0.001*	*NS*	*0.002*	*0.01*
Mental Health	Pre	72.3 (17.2)	81.6 (14.7)	78.8 (15.1)	69.4 (22.5)
	Post	78.2 (16.6)	85.2 (17.1)	85.6 (14.0)	79.7 (16.4)
	*P*	*0.01*	*NS*	*0.01*	*NS*
Role Emotional	Pre	59.8 (42.3)	75.2 (34.2)	82.3 (33.9)	68.1 (46.6)
	Post	70.5 (40.6)	83.3 (29.7)	88.9 (25.9)	78.3 (39.7)
	*P*	*NS*	*NS*	*NS*	*NS*

Preoperative and postoperative mean scores for each study group are shown for the eight dimensions assessed by the RAND-36 health-related quality of life questionnaire. Values are mean (standard deviation). *P*-values for statistically significant differences between preoperative and postoperative mean scores are shown for each surgical group. ^*^LCC = laparoscopic cholecystectomy, ^**^Pre = Preoperative mean score, ^***^Post = Postoperative mean score.

## Discussion

We studied health-related quality of life following four typical outpatient procedures at the time when patients had returned to work or comparable daily routines. Patients` self- assessed health profiles were documented pre- and postoperatively by the RAND-36 instrument. Surgical care significantly improved patients` experience of physical well-being and decreased bodily pain.

Overall, in the combined outpatient population a significant improvement was seen postoperatively in all dimensions of health-related quality of life. The finding that the general population reference scores were not reached in the relatively healthy study population by the time of the postoperative assessment probably indicates that rehabilitation was still going on. A study using EuroQoL 5-dimensional Classification Component Scores (EQ -5D) for assessment of health-related quality of life in outpatients did not find noticeable changes between preoperative and postoperative scores two weeks postoperatively [14,20]. Our choice of a health profile instrument may have been more sensitive in detecting changes than a preference-based measure [17,21]. So far there is no general agreement on the optimal instruments for evaluating recovery and outcome following ambulatory surgery [10,16]. Ideally such instruments should allow comparison of care across studies.

However, a procedure-specific approach seems more reasonable in the evaluation of outcome due to differences in the preoperative condition and postoperative recovery profiles in different types of surgical care. The surgical condition itself may have a significantly negative impact on quality of life preoperatively. Previous studies on patient-reported outcome following inguinal hernia repair have reported similarly low preoperative scores as seen in the present study [22]. In addition, significant improvements in all RAND-36/SF-36 dimensions were reported 6-months following inguinal hernia repair [22,23]. This overall improvement was not experienced by the inguinal hernia patients in present study, which is probably due to an earlier time point of preoperative assessment. The finding that postoperatively orthopedic patients still experienced more pain and reported more interference of physical health with working and daily routines than the two other study groups may also indicate that recovery was still going on at the time of the evaluation. A recent study compared patient-reported recovery profiles using an extended eight-item EQ5-D questionnaire and found a longer time of at least 3 months to full recovery following outpatient arthroscopic procedures compared to a time of one month following outpatient inguinal hernia repair or cosmetic breast augmentation surgery [16]. Slower recovery in the arthroscopic group was indicated by patient-reported surgical site-related disability.

Patients scheduled for ambulatory surgery may be healthier than the general population, which should be taken into account when analyzing the results. On the other hand, healthy patients may rate sudden changes in general health more incapacitating than patients with chronic health states [24]. In our patient cohort age and sex were not equally distributed in the study groups, and data cannot be generalized to apply to all day surgery patients undergoing the procedures in the present study. Surgical care itself may have a positive placebo effect on patients` perception of health [25]. We did not recruit a reference group of individuals not undergoing surgery. Instead, we used the general Finnish population reference values for comparison [18].

The present study describes patients self-assessed quality of life. We did not administer procedure-specific instruments for more detailed information of functional outcome in the different surgical groups, which is a limitation in the interpretation of the data.

The overall return rate (73%) of questionnaires in this study was satisfactory for a mail surveys in general [26]. Long sick leaves and forgetfulness may have influenced the low return rate (55%) in the shoulder surgery group. Although the RAND-36 questionnaire is comprised of several items, it is easy to administer and takes about 10 minutes to fill-in [17]. In this study completion of the questionnaires was satisfactory. Automated electronic communication by the internet or telephone text message services may be a more practical option for routine assessment of patient-reported outcome measures.

## Conclusions

Patient-reported outcome measures, including health-related quality of life are important indicators of quality of care and outcome. Assessment of the quality and length of recovery following specific procedures is necessary for recognition of potential areas of improvement in patient care and rehabilitation. In the present study the generic RAND - 36- instrument showed significant improvements postoperatively especially in physical health at the time when patients returned to work or comparable daily routines. Further studies are needed to determine the optimal practice in the assessment of quality of life following ambulatory surgery procedures.

## Competing interests

The authors do not have any competing interests.

## Authors’ contributions

KM, MH and ML contributed to study design, conduct of study and manuscript preparation. KM analyzed the data. All authors read and approved the final manuscript.

## Pre-publication history

The pre-publication history for this paper can be accessed here:

http://www.biomedcentral.com/1471-2253/12/30/prepub
